# Optical Coherence Tomography Parameters in Morbidly Obese Patients Who Underwent Laparoscopic Sleeve Gastrectomy

**DOI:** 10.1155/2016/5302368

**Published:** 2016-06-16

**Authors:** Berna Dogan, Ugur Dogan, Muhammet Kazim Erol, Mani Habibi, Nurullah Bulbuller

**Affiliations:** ^1^Department of Ophthalmology, Antalya Training and Research Hospital, 07100 Antalya, Turkey; ^2^Department of General Surgery, Antalya Training and Research Hospital, 07100 Antalya, Turkey; ^3^Department of General Surgery, Esenler Maternity and Child Health Hospital, 34230 Istanbul, Turkey; ^4^Department of General Surgery, Medicine Faculty, Akdeniz University, 07058 Antalya, Turkey

## Abstract

*Purpose*. To investigate changes in optical coherence tomography parameters in morbidly obese patients who had undergone laparoscopic sleeve gastrectomy (LSG).* Methods*. A total of 41 eyes of 41 morbidly obese patients (BMI ≥ 40) who had undergone LSG were included in study. The topographic optic disc parameters, central macular thickness (CMT), total macular volume (TMV), and retinal ganglion cell layer (RGCL) were measured by spectral-domain optical coherence tomography (SD-OCT). Subfoveal choroidal thickness (SFCT) was measured by enhanced deep imaging-optical coherence tomography (EDI-OCT).* Results*. The mean CMT was 237.4 ± 24.5 *μ*m, 239.3 ± 24.1 *μ*m, and 240.4 ± 24.5 *μ*m preoperatively, 3 months postoperatively, and 6 months postoperatively, respectively (*p* < 0.01). The mean TMV was 9.88 ± 0.52 mm^3^, 9.96 ± 0.56 mm^3^, and 9.99 ± 0.56 mm^3^ preoperatively, 3 months postoperatively, and 6 months postoperatively, respectively (*p* < 0.01). The mean RGCL was 81.2 ± 6.5 *μ*m, 82.7 ± 6.6 *μ*m, and 82.9 ± 6.5 *μ*m preoperatively, 3 months postoperatively, and 6 months postoperatively, respectively (*p* < 0.01). The mean SFCT was 309.8 ± 71.8 *μ*m, 331.0 ± 81.4 *μ*m, and 352.7 ± 81.4 *μ*m preoperatively, 3 months postoperatively, and 6 months postoperatively, respectively (*p* < 0.01). No statistically significant differences were found between the preoperative values and 3- and 6-month postoperative values in rim area (*p* = 0.34), disc area (*p* = 0.64), vertical cup/disc ratio (*p* = 0.39), cup volume (*p* = 0.08), or retinal nerve fiber layer (*p* = 0.90).* Conclusions*. Morbidly obese patients who undergo LSG experience a statistically significant increase in CMT, TMV, SFCT, and RGCL at 3 months and 6 months after surgery.

## 1. Introduction

Obesity is a major public health problem, with prevalence increasing at amazing rates in many countries. Genetic and environmental factors contribute to the development of obesity. Diagnosis of obesity and morbid obesity is based on determination of body mass index (BMI), which is calculated as weight/height^2^ (kg/m^2^). The World Health Organization (WHO) defines underweight as a BMI** ≤ **18.50, normal weight as BMI 18.50–24.99, overweight as BMI 25.00–29.99, obese class I as BMI 30.00–34.99, obese class II as BMI 35–39.99, and obese class III or morbidly obese as BMI** ≥ **40.

Bariatric surgery has become a very effective treatment option in the management of obesity. Laparoscopic sleeve gastrectomy (LSG), one of the foremost bariatric procedures, is believed to induce weight loss by physically restricting gastric capacity via removal of 80% or more of the stomach, including the fundus and greater curvature [1]. Gastric emptying and intestinal transit seem to be faster after LSG and exaggerated release of the satiety gut hormones. LSG might alter signalling from the gut to the hypothalamus and brainstem. Bariatric surgery reduce body weight by decreasing hunger, increasing satiation during a meal, changing food preferences, and increasing diet-induced energy expenditure. Manipulation of gastrointestinal anatomy through bariatric surgery has been shown to profoundly affect the physiological and metabolic processes that control body weight and glycaemia [2].

Obesity is highly associated with increased morbidity and early mortality due to the increased prevalence of chronic diseases, such as diabetes mellitus, cardiovascular disease, hypertension, stroke, and sleep apnea syndrome [3]. Although the effect of obesity on the eye has not been well documented, it has been associated with cataract, glaucoma, diabetic retinopathy, and age-related maculopathy [4].

Spectral-domain optical coherence tomography (SD-OCT) with faster scanning speed and higher spatial resolution has become essential clinical tools to diagnose and monitor retinal diseases. New-generation SD-OCT allows for early detection of subclinical retinal changes. The detection of such changes may provide a better understanding of the pathophysiological mechanisms of posterior segment eye diseases [5].

This is the first study to our knowledge which has investigated optical coherence tomography parameters in morbidly obese patients who undergo LSG. To clarify the relationship between optical coherence tomography parameters and obesity, we investigated the impact of weight loss on intraocular pressure (IOP), topographic optic disc parameters (retinal nerve fiber layer (RNFL), rim area (RA), disc area (DA), cup volume (CV), vertical cup/disc (C/D) ratio), central macular thickness (CMT), total macular volume (TMV), retinal ganglion cell layer (RGCL), and subfoveal choroidal thickness (SFCT) in morbidly obese patients who had undergone LSG.

## 2. Materials and Methods

### 2.1. Subjects

The study protocol was approved by the local Ethics Committee of Antalya Training and Research Hospital and conducted in accordance with the Declaration of Helsinki. Prior to initiation all subjects signed a detailed written consent form to confirm their understanding of the study procedures. The inclusion criteria for all subjects were best-corrected visual acuity of 20/20 or more, refractive errors between +2 D and −2 D spherical equivalent, primary diagnosis of morbid obesity (BMI ≥ 40), primary treatment of LSG for morbid obesity, and age ≥ 18. The exclusion criteria were morbid obesity with other endocrine disorders (e.g., diabetes mellitus or systemic arterial hypertension); cardiovascular disease or other serious chronic systemic diseases; history of smoking or alcohol consumption; history of ocular surgery, laser therapy, ocular trauma, or anterior or posterior segment disease; use of any medication within the previous 3 months; strabismus; amblyopia; IOP > 21 mmHg; or glaucomatous findings (e.g., glaucomatous optic disc changes or visual field defects). From 45 morbidly obese patients who had undergone LSG between May 2014 and May 2015 after diagnosis of morbid obesity, 41 were included and 4 were excluded, 1 due to glaucoma, 1 due to keratoconus, and 2 due to pseudotumor cerebri.

### 2.2. Measurement

The preoperative BMI and the 3- and 6-month postoperative BMI of the patients were calculated using the standard (WHO) formula (kg/m^2^). All patients underwent a detailed ophthalmic examination that included visual acuity testing, refraction assessment, anterior segment slit lamp biomicroscopy, fundus examination, and IOP measurement using a Goldmann applanation tonometer. Topographic optic disc parameters (RNFL, RA, DA, CV, and vertical C/D ratio), TMV, CMT, and RGCL assessment using SD-OCT (Cirrus HD OCT, Carl Zeiss Meditec, Dublin, CA, USA) preoperatively and at 3 and 6 months postoperatively. Subfoveal choroidal thickness (SFCT) measurements were performed by two experienced retinal specialists (Muhammet Kazim Erol and Berna Dogan) blind to the patients' BMI using a high-speed and high-resolution SD-OCT device. The participants were asked not to consume caffeine for at least 12 h before examination. Three consecutive measurements of SFCT were performed over 3 days and the average values were calculated. SFCT was measured perpendicularly from the outer edge of the retinal pigment epithelium to the choroid-sclera boundary at the fovea using a single line of 6 mm length centered horizontally on the fovea for visualization of the choroid. The TruTrack active eye tracking system, which enables the capture of multiple images in the same location, and the automatic real-time mean function, which combines these images, were used during each image acquisition. All measurements were performed between 9:00 and 11:00 AM to avoid diurnal fluctuations.

### 2.3. Statistical Analysis

As the sample size was smaller than 50, the Shapiro-Wilks test was performed to examine normal distribution. According to the timing of each measurement, the Friedman test was performed if the measurements were not normally distributed and repeated measures ANOVA was performed if they were normally distributed. If the difference between the measurements was significant, paired comparison was performed using the Bonferroni-Dunn test for nonparametric tests or the Bonferroni test for parametric tests. The correlations between continuous variables not displaying normal distribution were analyzed using the Spearman correlation test and the correlations between variables displaying normal distribution were analyzed using the Pearson correlation test. The level of significance was defined as *p* < 0.05. All analyses were conducted using the SPSS 22.0 software package (SPSS, Inc., Chicago, IL, USA).

## 3. Results

**Figure 1:**
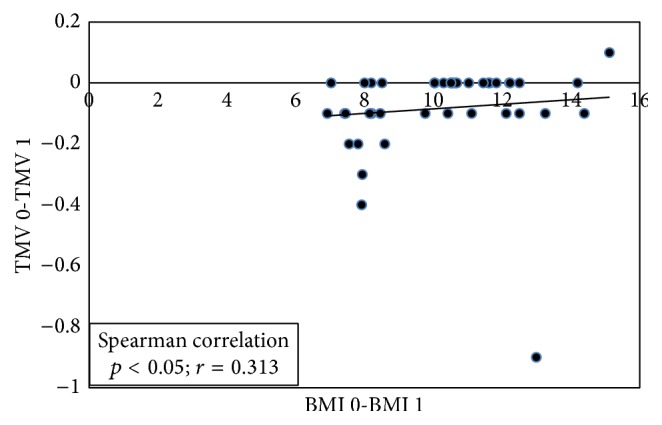
This figure shows that correlation between changes in total macular volume (TMV) and changes in body mass index (BMI). TMV 0: preoperative TMV; TMV 1: 3-month postoperative TMV; BMI 0: preoperative BMI; BMI 1: 3-month postoperative BMI.

The clinical characteristics of the study cohort and statistical findings are summarized in Table 1. The results of binary comparison of the parameters preoperatively and at 3 and 6 months postoperatively are shown in Table 2. The mean age of the 41 morbidly obese patients was 38.0 ± 8.6 years. A statistically significant decrease in BMI was detected at 3 months and 6 months postoperatively (*p* < 0.01). A statistically significant increase in CMT, TMV, SFCT, and RGCL was detected at 3 months and 6 months postoperatively (*p* < 0.01). There was significant correlation between changes in BMI and changes in TMV (*p* < 0.05, *r* = 0.313) (Figure 1). There was also significant correlation between changes in BMI and changes in SFCT (*p* < 0.04, *r* = 0.340). There was no significant difference between the women and the men in TMV, CMT, SFCT, and RGCL. No statistically significant differences were found between preoperative and 3- and 6-month postoperative RA (*p* = 0.34), DA (*p* = 0.64), vertical C/D ratio (*p* = 0.39), CV (*p* = 0.08), or RNFL (*p* = 0.90).

## 4. Discussion

Obesity results from morphological and functional changes in the adipose tissue associated with changes in various inflammatory, hormonal, and metabolic factors [3, 6]. Several possible pathophysiological mechanisms have been proposed to explain the association between morbid obesity and ocular diseases. Recent research has indicated that oxidative stress in obese individuals may increase as a result of hyperleptinemia, which may trigger pathological changes leading to elevated IOP. Obesity linked hyperleptinemia may cause oxidative damage to the trabecular meshwork [7–9]. Both the “mechanical” and “vascular” etiology theories of glaucoma may be related to obesity [4, 10]. With regard to the mechanical theory, obesity has been postulated to exert an effect on IOP by causing excessive intraorbital adipose tissue, increased episcleral venous pressure, and impairment of aqueous outflow facility [10, 11]. The vascular theory suggests that obesity has a significant effect on the human microcirculation [12], Stapleton et al. found that obese individuals have decreased nitric oxide (NO) levels, which could result in impaired dilatation of the vasculature [13]. Also, increased levels of some vasoconstrictor molecules such as endothelin-1 (ET-1) and angiotensin-II (Ang-II) have been reported to be associated with higher BMI [14]. ET-1 might be associated with the reduction of blood flow to the optical nerve head [15]. Impaired vascular supply to the optic nerve head is more predisposed to damage by elevated or normal IOP [4].

Several studies have found a significantly positive correlation between BMI and IOP [16–18]. These findings suggest that obesity is an independent risk factor for increase in IOP [16].

In our study, we found a statistically significant decrease in IOP at 3 months and 6 months postoperatively but no statistically significant differences between the topographic optic disc parameters (RNFL, RA, DA, and CV) preoperatively and at 3 and 6 months postoperatively.

Whereas choroidal blood flow is reduced through sympathetic efferent nerve activation and the release of noradrenalin, choroidal blood flow is increased through parasympathetic efferent nerve activation via NO signaling [19]. Identifying this role is significant in understanding the effect of obesity on CT, as lower levels of NO have been found in morbidly obese patients. This finding, together with the positive association identified between BMI and levels of vasoconstrictor molecules, explains how disruption of the vasodilator and vasoconstrictor balance as a result of morbid obesity can affect CT. Yilmaz et al. showed that BMI was negatively correlated with CT [20]. Erşan et al. found that the subfoveal CT was significantly thinner in obese children compared to nonobese children [21]. We found a statistically significant increase in SFCT at 3 months and 6 months postoperatively.

Bariatric surgery is the most successful treatment for significant weight loss, resolution of type 2 diabetes, and the prevention of future weight gain [1]. The impact of weight change (weight gain or loss) on eye diseases has not been well documented [4].

Brynskov et al. showed that patients with type 2 diabetes who underwent bariatric surgery had clinically stable diabetic retinopathy in the first postoperative year and a clinically negligible but statistically significant foveal thickening 6 months postoperatively [22]. Erşan et al. found that the macula thickness was significantly thinner in obese children compared to nonobese children [21].

In our study, morbidly obese patients who undergo LSG experience a statistically significant increase in central macular thickness, total macular volume at 3 months and 6 months postoperatively. There was no significant difference between the women and the men in total macular volume, central macular thickness.

In the macula, the area that contains the highest carotenoid concentration within the body the macular pigment is composed of lutein and zeaxanthin [23]. Mainly present in the nerve fiber layers and RGCL, with peak concentrations in the fovea [24], the macular pigment is thought to function as a blue-light filter and antioxidant that enhances visual acuity by decreasing chromatic aberration, reduces visual discomfort by attenuating glare and dazzle, enhances detail by absorbing “blue haze,” and increases visual contrast. By doing so, macular pigment protects the retina from damaging influences believed to play a role in the pathogenesis of age-related macular degeneration [24, 25].

Obese subjects tend to have lower concentrations of retinal lutein and zeaxanthin. Adipose tissue could compete with the retina for uptake of lutein and zeaxanthin, resulting in less incorporation into the retina and lower macular pigment optical density (MPOD) [26]. Hammond et al. found that both men and women with a body fat percentage >27% had a 16% lower MPOD compared to subjects with a body fat percentage ≤27% and identified an inverse relationship between MPOD and both BMI and the percentage of body fat. Given that up to 80% of total carotenoids in the human body are stored in body fat, a higher body fat content and BMI may be expected to influence the quantities of lutein and zeaxanthin in the retina [27]. However, the precise relationship between both body fat and BMI and retinal carotenoid concentration remains unclear.

In a rat retinal model, Zhang et al. found that lutein increased the number of RGCs after N-methyl-D-aspartate- (NMDA-) induced retinal damage [28]. Lutein is an antiapoptotic, direct free-radical scavenger that prevents macular damage and plays a neuroprotective role by decreasing oxidative stress; it is unclear whether lutein directly protects RGCs [29]. In another study of this relationship, Van Der Veen et al. found a significantly positive correlation between MPOD and central foveal thickness [24]. Supporting this finding, Liew et al. found a significantly positive relationship between MPOD and central retinal thickness as measured by OCT [30].

There are several limitations of the current study. Firstly, it is a single-center study and has a relatively small sample size. Secondly, we obtained SFCT measurements manually using EDI-OCT, which we believe that it can cause errors in measurement. To help overcome this limitation, SFCT measurements were performed by two experienced retinal specialists blind to the patients' BMI. Three consecutive measurements of SFCT were performed over 3 days and the average values were calculated. Another potential limitation is the lack of macular pigment optical density investigation that might support our findings.

## 5. Conclusions

Morbidly obese patients who undergo LSG experience a statistically significant increase in central macular thickness, total macular volume, subfoveal choroidal thickness, and retinal ganglion cell layer at 3 months and 6 months postoperatively. No statistically significant differences were found between preoperative and 3- and 6-month postoperative topographic optic disc parameters. The efficacy of obesity treatment in reducing the risk of eye diseases is unknown. Therefore, experimental, cellular, or molecular studies may expand our understanding of the impact of obesity on eye.

## Figures and Tables

**Table 1:** Preoperative and 3-month and 6-month postoperative values.

Parameter	Preoperative	3-month postoperative	6-month postoperative	*p* value
Body mass index (kg/m^2^)	47.9 ± 4.5	37.6 ± 4.4	31.7 ± 3.6	<0.01^1^
Intraocular pressure (mmHg)	17.8 ± 2.6	16.3 ± 2.1	15.6 ± 2.0	<0.01^2^
Retinal nerve fiber layer (*μ*m)	93.4 ± 12.3	93.6 ± 11.8	93.5 ± 11.6	0.90^2^
Rimarea (mm^2^)	1.47 ± 0.28	1.47 ± 0.26	1.48 ± 0.28	0.34^2^
Discarea (mm^2^)	1.75 ± 0.29	1.74 ± 0.31	1.75 ± 0.31	0.64^2^
Cup/disk vertical ratio	0.34 ± 0.17	0.35 ± 0.17	0.35 ± 0.17	0.39^1^
Cup volume (mm^3^)	0.076 ± 0.085	0.078 ± 0.083	0.077 ± 0.085	0.08^1^
Central macular thickness (*μ*m)	237.4 ± 24.5	239.3 ± 24.1	240.4 ± 24.5	<0.01^2^
Total macular volume (mm^3^)	9.88 ± 0.52	9.96 ± 0.56	9.99 ± 0.56	<0.01^2^
Subfoveal choroidal thickness (*μ*m)	309.8 ± 71.8	331.0 ± 81.4	352.7 ± 81.4	<0.01^2^
Retinal ganglion cell layer (*µ*m)	81.2 ± 6.5	82.7 ± 6.6	82.9 ± 6.5	<0.01^2^

Data are mean ± SD.

^1^Repeated measures ANOVA test; ^2^Friedman test.

**Table 2:** Binary comparison of preoperative and postoperative 3-month and 6-month values.

	Comparison of preoperative and 3-month postoperative	Comparison of preoperative and 6-month postoperative	Comparison of 3-month and 6-month postoperative
Body mass index^1^	<0.01	<0.01	<0.01
Intraocular pressure^2^	<0.01	<0.01	<0.01
Central macular thickness^2^	0.01	<0.01	0.02
Total macular volume^2^	0.01	<0.01	0.04
Subfoveal choroidal thickness^2^	<0.01	<0.01	<0.01
Retinal ganglion cell^2^	<0.01	<0.01	0.28

^1^Repeated measures ANOVA; ^2^Friedman test.
